# Derrone induces autophagic cell death through induction of ROS and ERK in A549 cells

**DOI:** 10.1371/journal.pone.0218659

**Published:** 2019-06-19

**Authors:** Myung-Ji Kang, Soo-Yeon Kim, Eun-Bin Kwon, Yang Hee Jo, Mi Kyeong Lee, Hyun-Sun Lee, Dong-Oh Moon, Mun-Ock Kim

**Affiliations:** 1 Natural Medicine Research Center, Korea Research Institute of Bioscience and Biotechnology (KRIBB), Cheongju, Chungbuk, Republic of Korea; 2 College of Pharmacy, Chungbuk National University, Cheongju, Chungbuk, Republic of Korea; 3 Department of Biology Education, Daegu University, Gyeongsan, Gyeongsangbuk, Republic of Korea; Sechenov First Medical University, RUSSIAN FEDERATION

## Abstract

We studied the effect of derrone (DR), one of the major compounds of unripe fruits of *Cudrania tricuspidata*, on cancer cell death. DR inhibited cell growth of various cancer cells, and that was partially associated with apoptosis in A549 cells. DR showed the autophagic features, such as the conversion of LC3-I to LC3-II, the formation of autophagosome and the downregulation of SQSTM1/p62 (p62). The treatment of autophagy inhibitor reversed DR-mediated cell death, suggesting that DR induces autophagic cell death. The increase of cytoplasmic Ca^2+^ and ROS by DR treatment significantly influences the formation of autophagosomes; however, only ROS scavengers significantly rescued the reduced cell viability. Additional results revealed that treatment of DR induces sustained phosphorylation of ERK and the inhibition of ERK phosphorylation using U0126 (ERK inhibitor) markedly attenuated DR-induced cell death. Overall, these results suggest that DR induces autophagic cell death through intracellular ROS and sustained ERK phosphorylation in A549 cells.

## Introduction

Despite the improvement in many anticancer drugs, the mortality rate of lung cancer is still increasing [1]. Lung cancer is classified into small cell lung cancer (SCLC) and non-small cell lung cancer (NSCLC) according to the size and histology of cancer cells. NSCLC accounts for approximately 80% of the lung cancer [2]. According to the stage, treatment with NSCLC is attempted with radiation therapy, chemotherapy, target treatment, and bronchoscopy. Since the early 2000s, the development of target therapies that selectively attack cancer cells has been activated, and lung cancer target treatment drugs such as gefitinib (Iressa) and erlotinib (Tarceva) have been used [3–5]. Because the target therapies have problems of high cost and low stability, patients receiving treatment were limited. Therefore, many scientists continue to make efforts to develop new lung cancer drugs.

Autophagy and apoptosis play an important role in NSCLC progression. Autophagy is activated in response to several cellular stresses, the process delivers cytoplasmic material and organelles to lysosomes *via* double membrane vesicles called autophagosomes for degradation. The formation of autophagosomes is controlled by a specific set of autophagy genes called atg genes. ATG8 (in mammals LC3), a well-established marker of autophagy, is covalently linked to phosphatidylethanolamine on the autophagic membrane during autophagosome formation. Apoptosis is usually characterized by membrane blebbing, cytoplasmic shrinkage, mitochondrial depolarization, release of apoptotic factors from the mitochondria, DNA fragmentation and apoptotic body formation [6].

Autophagy can play a positive and negative role in promoting apoptosis in NSCLC. Autophagy is always suppressed by several oncoproteins, such as PI3K, AKT, Bcl-2 and mutant p53, which may prevent excessive protein degradation in starved or stressed tumor cells [7, 8]. On the other hand, persistent activation of autophagy causes autophagic programmed cell death or apoptosis [9, 10].

*Cudrania tricuspidata* (Moraceae) is a deciduous tree which is cultivated in China, Japan and Korea. The roots, stems, barks and fruits of *C*. *tricuspidata* have been widely used as traditional medicines and various pharmacological efficacy including anti-atherosclerotic, anti-inflammatory, anti-fungal, anti-lipid peroxidation, anti-oxidant effect have been studied [11–15]. Among them, fruits of *C*. *tricuspidata* have been reported to contain diverse active constituents such as polyphenols, isoflavonoids and flavonoids [16, 17], which were affected by environmental conditions including maturation stages. Recently, we investigated the chemical compositions and anti-obesity effects of unripe and ripe fruits of *C*. *tricuspidata* [18]. Further study on the chemical constituents of *C*. *tricuspiata* found that derron (DR), an isoflavonoids from unripe fruit, inhibited cell growth of A549 cells (derived from NSCLC). In this study, we investigated molecular mechanisms involved in DR-induced cell death, focusing on autophagy and apoptosis in A549 cells.

## Materials and methods

### Reagent and materials

Chloroquine (CQ), *N*-acetyl-L-cysteine (NAC) and acridine orange hemi (Zinc chloride) salt were purchased from Sigma-Aldrich (Saint Louis, MO, USA). Caspase-8,-9, or -3 colorimetric assay kit and Z-VAD-FMK (a pan-caspase inhibitor) were obtained from R&D systems (MA, USA). Cycletest Plus DNA kit and FITC-Annexin V were purchased from BD bioscience Pharmingen (San Jose, CA, USA). Wortmannin and U0126 were purchased TOCRIS (Bristol, UK). 2’7’-dichlorofluorescein diacetate, BAPTA-AM, Ru360 and JC-1 were obtained from Calbiochem (San Diego, CA, USA). Dihydroethidium, lipofectamine 2000, anti-Alexa Fluor 488, Fluo4-AM and BAPTA were purchased from Invitrogen (Carlsbad, CA, USA). Rhod2 and Ruthenium Red were obtained from Abcam (Cambridge, UK). The antibodies used in this study are as follows; anti-caspase-3, anti-caspase-9, anti-caspase-8, anti-PARP, anti-ATG5, anti-total ERK, anti-p-ERK (Cell signaling Technology, MA, USA), LC3B/MAP1LC3B (NOVUS Biologicals, USA), anti-p62 lck ligand (BD bioscience Pharmingen, CA, USA), HRP-conjugated anti-rabbit IgG and HRP-conjugated anti-mouse IgG (Santa Cruz Biotechnologies, CA, USA).

### Isolation of DR

*C*. *tricuspidata* unripe fruits were collected from the herb garden at Chungbuk National University from May 2013. A voucher specimen (CBNU2013-CTUF) was deposited at the herbarium of the College of Pharmacy, Chungbuk National University. The unripe fruits (556.0 g) were extracted 2 times with 100% MeOH at room temperature, which yielded the MeOH extract (20.4 g). The MeOH extract was suspended in H_2_O, then partitioned successively with solvents of rising polarity, to obtain *n*-hexane (0.8 L), CH_2_Cl_2_ (0.7 L), EtOAc (0.8 L), and *n*-BuOH (0.8 L). The CH_2_Cl_2_ fraction (CTUM, 3.3 g) was subjected to column chromatography over Sephadex LH-20 eluted with 100% MeOH to give four subfractions (CTUM1-CTUM4). Derrone (111.1 mg) was isolated from CTUM3 by semi-preparative HPLC eluted with MeCN-H_2_O (57:43) [18]. Derrone is light brown amorphous syrup; UV (MeOH) λ_max_: 266 nm; ESI-MS *m/z* 337 [M+H]^+^; ^1^H-NMR (methanol-*d*_4_, 500 MHz) (see S1 Table).

### Cell culture

Human lung cancer A549 cells, human mucoepidermoid pulmonary carcinoma H292 cells and human prostate cancer PC3 cells were obtained from American Type Culture Collection (ATCC, Manassas, VA, USA). Cell were cultured in RPMI-1640 (Welgene, Korea) medium, supplemented with 10% fetal bovine serum (FBS, Gibco, USA) and 1% penicillin-streptomycin (Gibco, USA) at 37°C, 100% humidity and 5% CO_2_. Human colorectal carcinoma HCT116 cells were obtained Bioevaluation Center (Korea Research Institute of Bioscience & Biotechnology, Republic of Korea). HCT116 cells were cultured in high glucose-Dulbecco’s Modified Eagle Medium (DMEM, Welgene, Korea) containing 10% (v/v) heat-inactivated FBS and 1% penicillin-streptomycin at 37°C in a humidified atmosphere of 5% CO_2_ in air.

### Cell viability

Cells (1×10^5^ cells/ml) were grown in 24-well plates for 24 h and then were treated with indicated concentrations of DR for designated incubation times. After incubation, cells were treated with MTT solution (final 0.5 mg/ml) for 30 min. Purple formazan was dissolved in dimethyl sulfoxide (DMSO) and absorbance was read at 540 nm using ELISA plate reader (Epoch, Bioteck, USA).

### Immunoblotting

Cell were washed with ice-cold PBS and lysed with lysis buffer (Pro-Prep, iNtRON) containing protease inhibitor in ice for 30 min. After centrifugation (13,200 rpm, for 25 min at 4°C), collected supernatants were quantified using the Bredford method. The lysates boiling for 5 min at 95°C, separated by SDS-PAGE gels and transferred to a polyvinylidene difluoride membranes (PVDF, Millipore, MA, USA). After blocking nonspecific binding sites for 30 min by Ez-Block Chemi (Amherst, USA), membrane was incubated with specific primary and secondary antibodies. Membrane was then washed three times TBS-T for 30 min. Enhanced chemiluminescence (ECL, Thermo, USA) detection was used to detect immune complexes signal. Equal amount of proteins was assessed by anti-tubulin as internal controls. Protein band intensity was measured by densitometric analysis using the NIH Image J program (National Institutes of Health, Bethesda, MD, USA).

### Measurement of intracellular calcium

A549 cells were harvested and incubated with 1 μg/ml Fluo-4 AM or 1 μg/ml Rhod 2-AM at 37°C for 15 min, washed with HBSS (without Ca^2+^ or Mg^2+^). Then immediately analyzed on a flow cytometer (FACSCalibur, Becton Dickinson, CA, USA) using FL-1 or FL-2 channel.

### Transmission electron microscopy

A549 cells (2×10^5^ cells/ml) were grown in 100 mm plates for 24 h and then were treated with 60 μM DR. Cell were collected and prefixed primary fixing solution (2.5% paraformaldehyde, 2.5% glutaraldehyde). After staining with uranyl acetate and sections were visualized under electron microscope (CM20T, Philips, Netherlands).

### Analysis of cell cycle and Annexin V staining

A549 cells (2×10^5^ cells/ml) were grown in 12-well plates for 24 h and then were treated with various concentrations of DR for 24 h. Cells were prepared using the Cycletest Plus DNA kit according to the manufacturer's instructions and flow cytometric analysis for cell cycle analysis was performed. Annexin V-FITC staining was performed according to the manufacturer's instructions using the Apoptosis Detection Kit and analyzed via the FL-1 channel of a flow cytometer.

### Flow cytometric detection of ROS

Intracellular ROS levels were measured flow cytometry in cells loaded with the redox-sensitive dye dihydroethidium (HE) and 2’7’-dichlorofluorescein diacetate (H_2_DCF-DA). Approximately 5×10^5^ cells were harvested by trypsinization, resuspended in 0.5 ml PBS and incubated with 1 μM HE or 5 μM H_2_DCF-DA for 20 min in the dark at 37°C. Fluorescence was recorded on FL-2 channel for HE and FL-1 channel for DCF using a flow cytometer.

### Caspase activity assay

Total colorimetric caspase 3, 8, 9 assay were performed using commercial kit according to the manufacturer’s instructions. Briefly, cell were cultured O/N in 60 mm dishes, and then treated with various concentrations of DR (0, 20, 40, 60 and 80 μM) for 24 h. Cell were lysed with lysis buffer on ice for 30 min and then centrifuged at 13,200 rpm for 15 min. Equal amount of protein (50 μg) from each sample was added to the reaction mixture containing each substrate (DEVE-pNA, IETD-AFC or LEHD-pNA) and incubation for 120 min at 37°C. The absorbance was read at 405 nm in a micro plate reader. Each sample for the assay was taken in duplicates.

### Immunocytochemistry

A549 cells were cultured on cover slips and fixed with 4% paraformaldehyde in PBS for 10 min at room temperature. After washing at three times with PBS, the fixed cells were permeabilized with 0.1% Triton-X 100 for 5 min at RT. After three times washes with PBS, the cells were incubated with blocking solution (5% BSA in PBS) for 30 min and then with primary antibody overnight at 4°C. The next day, the cells were washed with PBS and then incubated with secondary antibody for 1h on the rocker. The cells were washed with PBS and Hoechst 33342 stained for 30 min. After washing, the cover slips were mounted on slide using mounting media. Cells were visualized with a fluorescence microscope (ZEISS, Germany).

### Statistical analysis

Data are presented as mean± standard deviation (SD). Statistical analysis was performed using Student's *t*-test for the *in vitro* experiments. Differences were considered significant at *p*<0.05 (*), *p*< 0.01 (**), and *p*< 0.001 (***).

## Results

### DR decreases cell growth of various cancer cells

**Fig 1:**
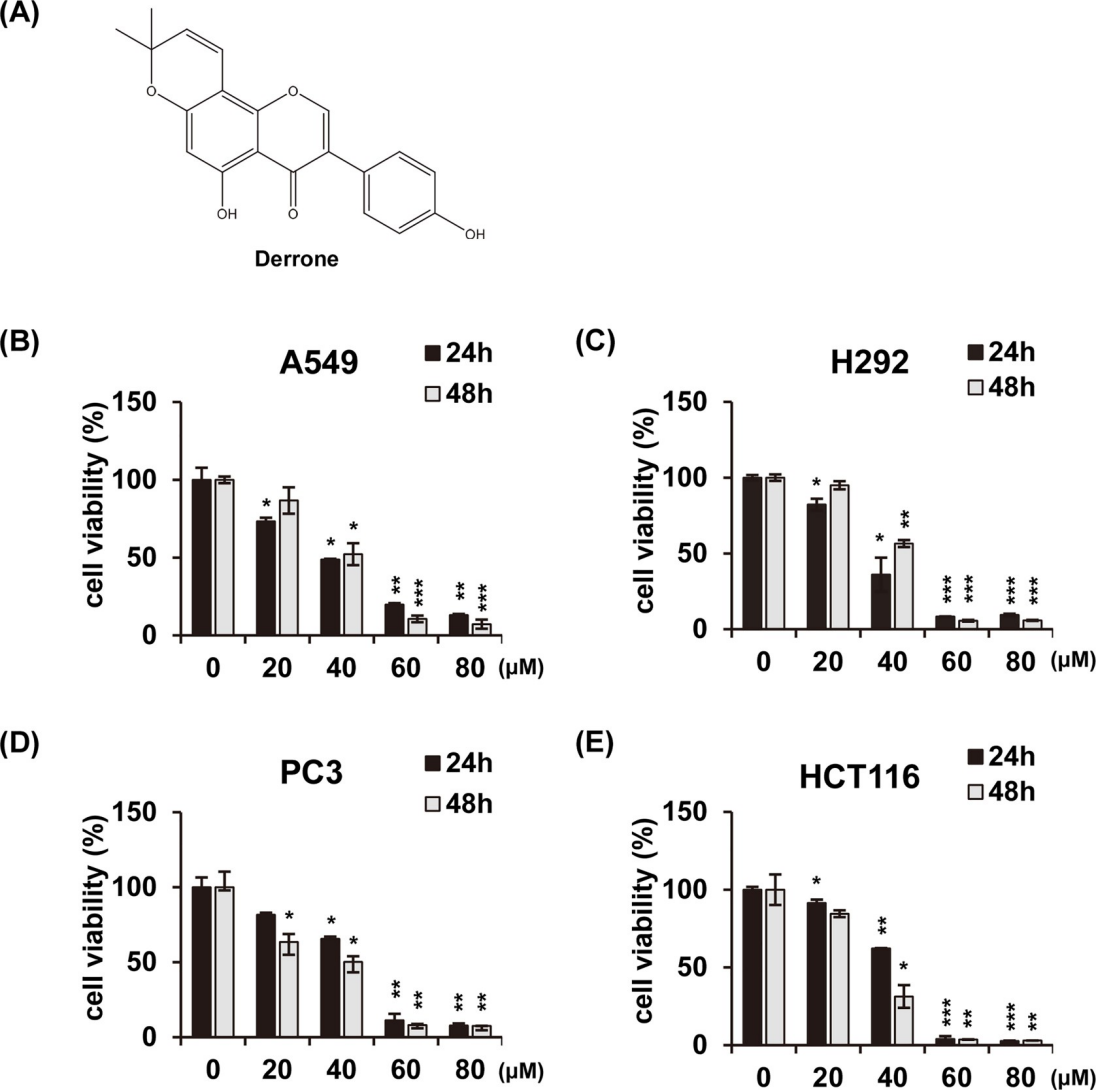
The molecular structure of DR and its cytotoxic effects on various cancer cell lines. (A) The molecular structures of the DR. Non-small cell lung cancer A549 cells (B), mucoepidermoid pulmonary cancer H292 cells (C), prostate cancer PC3 cells (D), and colon cancer HCT116 cells (E) were treated with the DR of indicated concentrations for 24 h or 48 h, and then measured by MTT assay. All data are expressed as the mean ± SD obtained from at least three independent experiments. Differences were considered significant at *p*<0.05 (*), *p*< 0.01 (**), and *p*< 0.001 (***) compared with the DMSO control.

To investigate the effect of DR on cell growth, DR was treated with different doses in various cancer cell lines (A549, human lung adenocarcinoma epithelial cells; H292, human lung mucoepidermoid cells; PC3, human prostate cancer cells; and HCT116, human colon cancer cells) and analyzed for cell viability using MTT assay (Fig 1B–1E). DR dissolved in DMSO and made 1000× stock with 80, 60, 40, and 20 mM in series. The solvent is finally diluted 1000-fold with the cell treatment. In all experiments, we treated all controls with 0.1% DMSO. DR inhibited cell growth in a concentration-dependent manner without any specificity for the four cell lines with different histological origins. The IC_50_ values of DR against A549, H292, PC3 and HCT116 were 42.7 μM, 39.3 μM, 45.0 μM and 42.4 μM, respectively. Collectively, these results indicate that DR reduces cell growth that are not limited to specific cancer cell lines.

### DR-mediated cell death is partially involved in apoptosis

**Fig 2:**
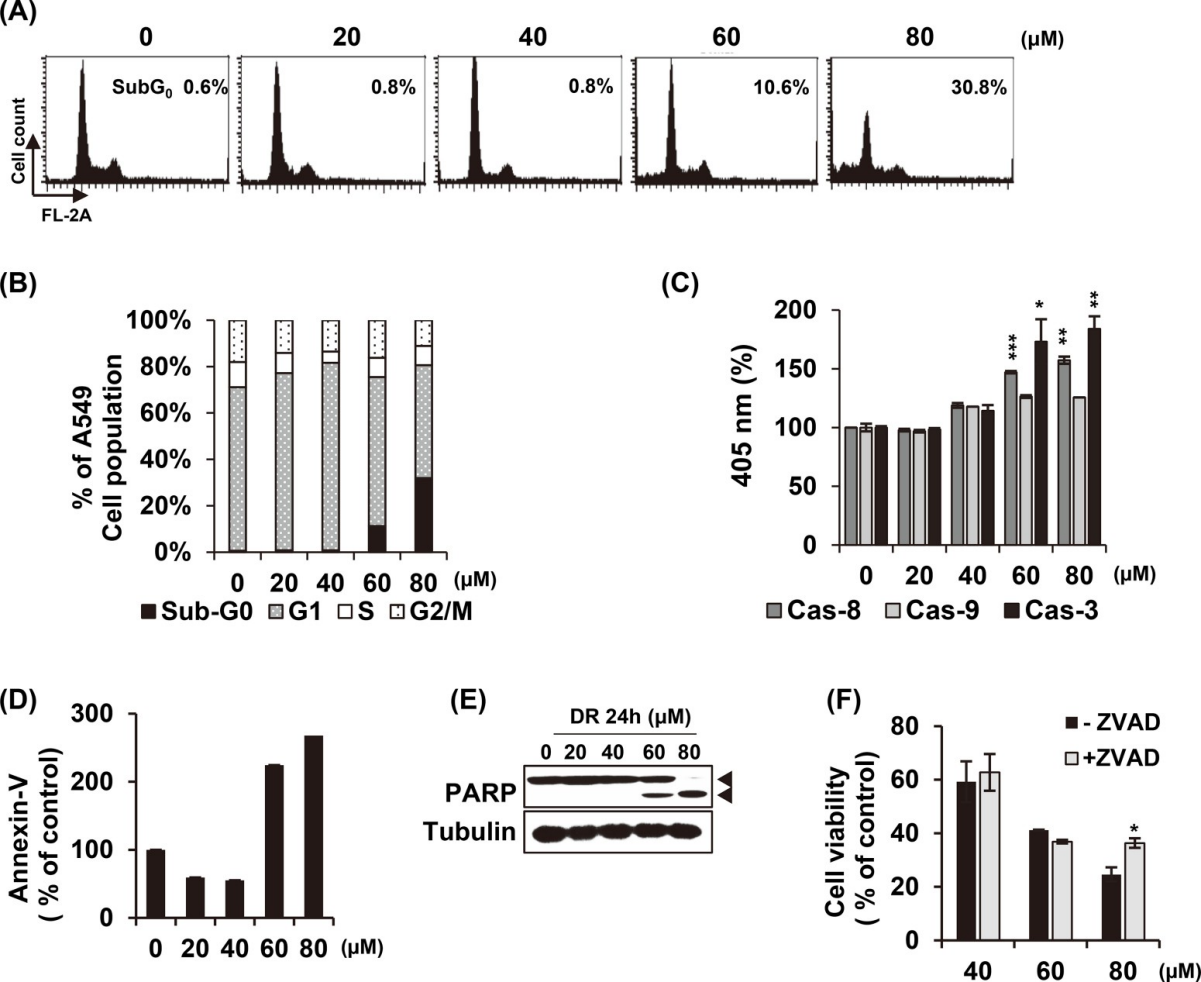
DR induces non-apoptotic cell death at low concentrations. (A) DR was treated with various concentration for 24 h, and cell cycle analysis was performed using flow cytometry. (B) The cell cycle distribution was quantified and represented as a bar graph. (C) After 24 h incubation with the indicated concentrations of DR, the A549 cells were lysed and aliquots were assayed for *in vitro* caspase-8, - 9 and -3 activity. (D) After DR treatment for 24 h, the cells were stained with Annexin V. Early apoptotic Annexin V-positive cells were detected by flow cytometry. (E) After treatment of DR with the indicated concentrations, the cells were lysated and analyzed by western blotting. (F) Cells were co-treated with pan caspase inhibitor (_Z_-VAD-fmk, 20 μM) and cell viability were measured by MTT assay. Statistical differences were presented p<0.05 (*), p<0.01 (**), and p<0.001 (***) compared with the DR alone; p<0.01 (^##^) compared with the DMSO control.

To investigate whether the inhibition of cell growth by DR treatment was caused by apoptosis, we examined the changes of apoptotic markers in A549 cells after treatment with DR. Cell cycle analysis showed a gradual increase in the apoptotic sub-G1 phase at a concentration of 60 μM and 80 μM DR for 24 h without arresting at specific cell cycle phases (Fig 2A and 2B). There are two major apoptosis signaling pathways: the death receptor (extrinsic) pathway and the mitochondrial (intrinsic) pathway. Caspases, a family of cysteine proteases, plays an essential role in both pathways. Death receptor pathway is initiated by caspase-8, and activation of the mitochondrial apoptotic pathway leads to activation of caspase-9. The death receptor and the mitochondrial pathway ultimately activate the cell death executor caspase-3. We treated A549 cells with various concentrations of DR for 24 h and confirmed the proteolytic activity of caspase-8, -9 and -3 (Fig 2C). Similar to the results of the cell cycle analysis, the activity of caspases was significantly increased in the DR 60 μM and 80 μM treatment groups. Annexin-V staining enables the identification of cells with deteriorated membrane integrity at an early apoptotic stage. As shown in Fig 2D, Annexin V-positive cells were markedly increased in cells treated with DR from 60 μM. Fig 2E presented the gradual cleavage of Poly(ADP-ribose) polymerase-1 (PARP-1) from 60 μM to 80 μM of DR. PARP-1 is proteolysed by caspases during the execution of the apoptotic process. Finally, pretreatment of z-VAD-fmk, a pan-caspase inhibitor, significantly reversed the cell death by 80 μM DR treatment but did not restored by 60 μM (Fig 2F). These results suggest that DR causes apoptotic markers to appear, but based on the fact that cell death is not restored by caspase inhibitor apoptosis, DR-induced A549 cell death is associated with non-apoptotic cell death mode can be involved.

### Autophagy is another cause of DR-induced cell death

**Fig 3:**
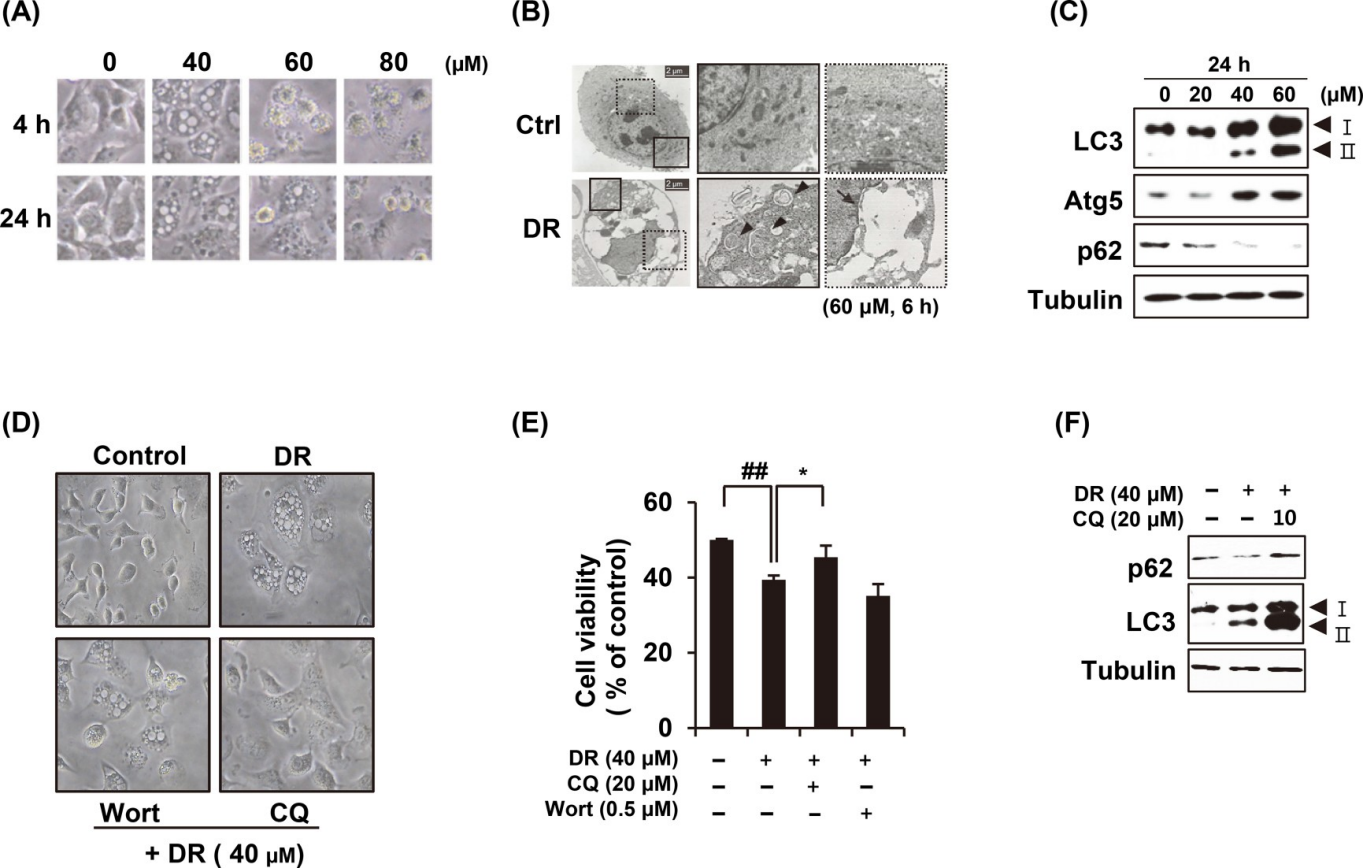
DR induced autophagy in A549 cells. (A) After A549 cells were treated with DR, the morphological change of cells was observed under the microscope. (B) Cells were treated with 60 μM DR for 6 h and observed under transmission electron microscopy. Arrowheads indicate autophagosome and arrows denote the vacuoles. (C) Cells were treated various concentrations of DR for 24 h before the western blot analysis. (D) Cells were treated 40 μM DR for 24 h with or without 1 h pretreatment with 0.5 μM wortmannin (Wort) or 20 μM chloroquine (CQ) and the morphological change of cells was observed under the microscope. (E) Cell viability was measured by MTT assay. Statistical differences were presented p<0.05 (*) compared with the DR alone; p<0.01 (^##^) compared with the DMSO control. (F) Cells were treated DR for 24h and immunoblot analysis was performed after cell lysis.

After A549 cells were treated with various concentrations of DR, morphological changes were observed under a microscope. Cytoplasmic vacuoles were noticeable from 4 h after treatment of 40 μM DR. In the cells treated with 80 μM, cytoplasmic contraction, a morphological feature of typical apoptosis, was observed at 4 h and most of the cells were floating at 24 h (Fig 3A). To determine the origin of cytoplasmic vacuoles, we enlarged the cell using transmission electron microscopy (Fig 3B). In the DR-treated group, the intracellular debris in the closed double membrane, which appeared to be autophagosomes were observed (Fig 3B, arrow head). In addition, the vacuoles in which all contents are empty are thought to be fused together after autolysosome formation (Fig 3B, arrow with dotted line). Immunoblot analysis carried out to confirm the expression of autophagy-related marker proteins such as LC3, ATG5 and p62. The conversion of LC3-I to LC3-II and expression of ATG5 were increased after 6 h of 40 μM DR treatment, whereas p62 was decreased (Fig 3C). We further tested whether autophagy inhibitors could blocked the formation of vacuoles. Chloroquine is a lysosomotropic agent that inhibits endosomal acidification and blocks autolysosome formation. Wortmannin is a class III PI3-kinase inhibitor that blocks autophagy at the upstream stage and reduces the conversion of LC3-I to LC3-II. Pretreatment of chloroquine inhibited DR-induced cellular vacuolation, whereas wortmannin did not (Fig 3D). Chloroquine significantly rescued the cell viability inhibited by DR (Fig 3E). Chloroquine pretreatment also restored DR-induced p62 degradation, while the conversion of LC3-I to LC3-II was more increased in A549 cells (Fig 3F). This result shows that DR-induced autophagosomes was inhibited the binding of lysosome by treating chloroquine. Collectively, we suggest that DR induces macroautophagy in A549 cells, which contributes to cell death.

### DR induces intracellular ROS generation

**Fig 4:**
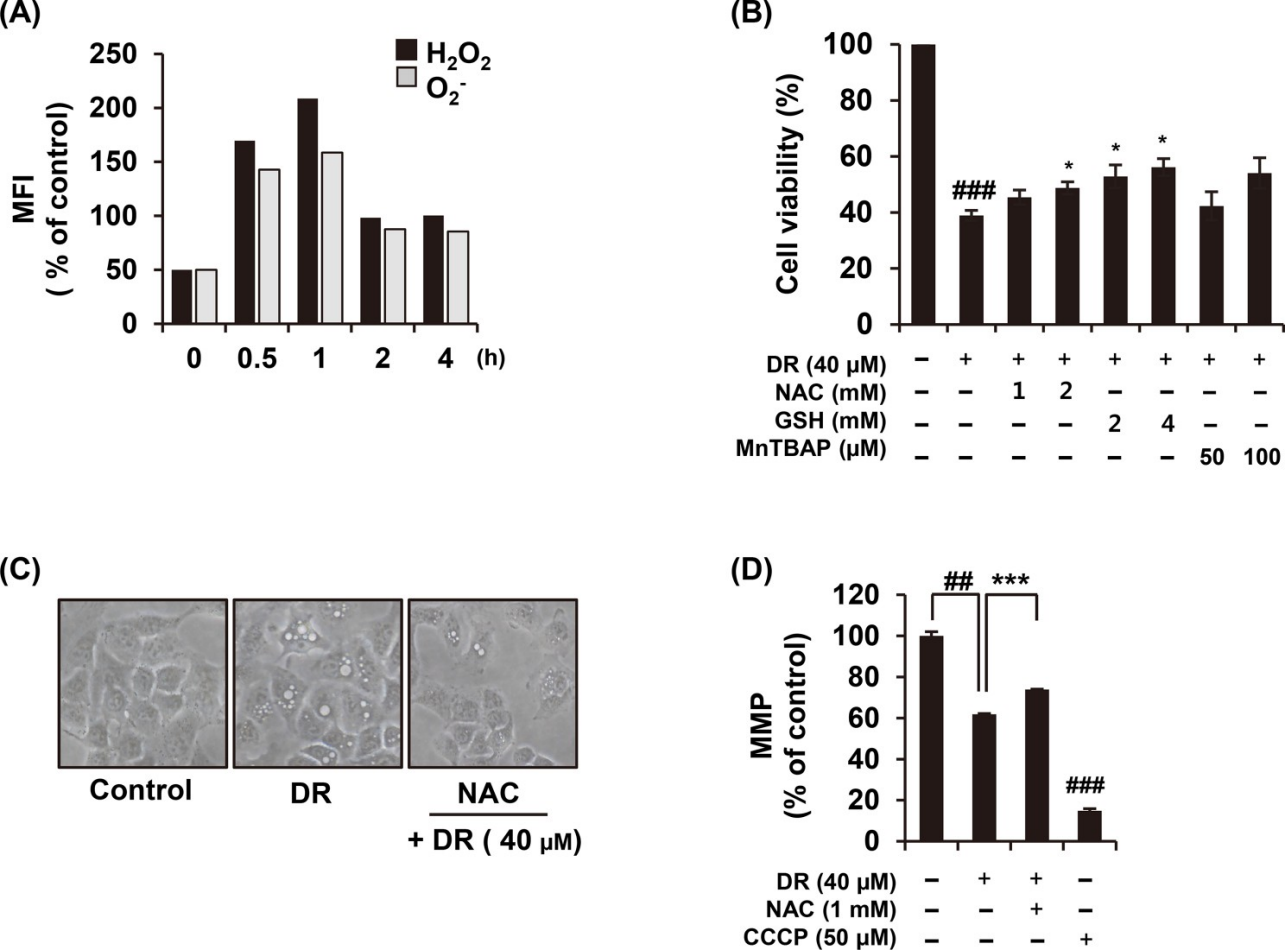
Increased ROS production by DR treatment is related to autophagy. (A) After A549 cells were treated with DR, the cells were loaded with the hydrogen peroxide sensitive dye H_2_DCFDA or the superoxide sensitive dye HE at 37°C for 30 min, and ROS generation was then measured using flow cytometry. (B) Cells were treated 40 μM DR for 24 h with or without treatment with NAC, GSH, and MnTBAP. The cell viability was measured by WST assay. (C) The morphological change of cells was observed under the microscope. (D) The cells were incubated with DiOC_6_(3) for 30 min 37°C in the dark, and analyzed by a flow cytometery. Carbonyl cyanide 3-chlorophenylhydrazone (CCCP; 40 nM) was used as a positive control. Statistical differences were presented p<0.05 (*), p<0.01 (**), and p<0.001 (***) compared with the DR alone; p<0.01 (##) compared with the DMSO control.

Mounting evidences suggested that ROS could be a factor in activating the autophagy pathway [19, 20]. As shown in Fig 4A, treatment with 40 μM DR stimulated ROS production, showing a sharp increase up to 1 h, and thereafter decrease. In order to confirm that DR-induced ROS is associated with autophagy induction, the cells were pre-treated with ROS scavenger, an *N*-acetyl-L-cysteine (NAC), before treatment of DR. The pretreatment of NAC completely abolished the macroautophagic (visible) vacuoles produced by DR (Fig 4C). We examined the effect of ROS on DR-mediated cell growth inhibition with NAC, glutathione or MnTBAP. Compared to NAC pretreatment, the glutathione (GSH) pretreatment group was more significantly restored by DR-induced cell death. MnTBAP, a superoxide anion scavenger, also showed a tendency to restore cell death by DR. Based on these results, we confirmed that DR-induced cell death in A549 cells is closely related to intracellular ROS production (Fig 4B). In addition, ROS induces autophagy accompanied by a strong mitochondrial dysfunction [21]. Pretreatment of NAC effectively recovered mitochondrial membrane potential (MMP, *Δ*Ψm) during DR treatment (Fig 4D). Collectively, these results indicate that DR increases intracellular ROS production and mitochondrial dysfunction, and that ROS is a factor in DR-mediated autophagic cell death.

### DR increases free cytoplasmic Ca^2+^ concentration

**Fig 5:**
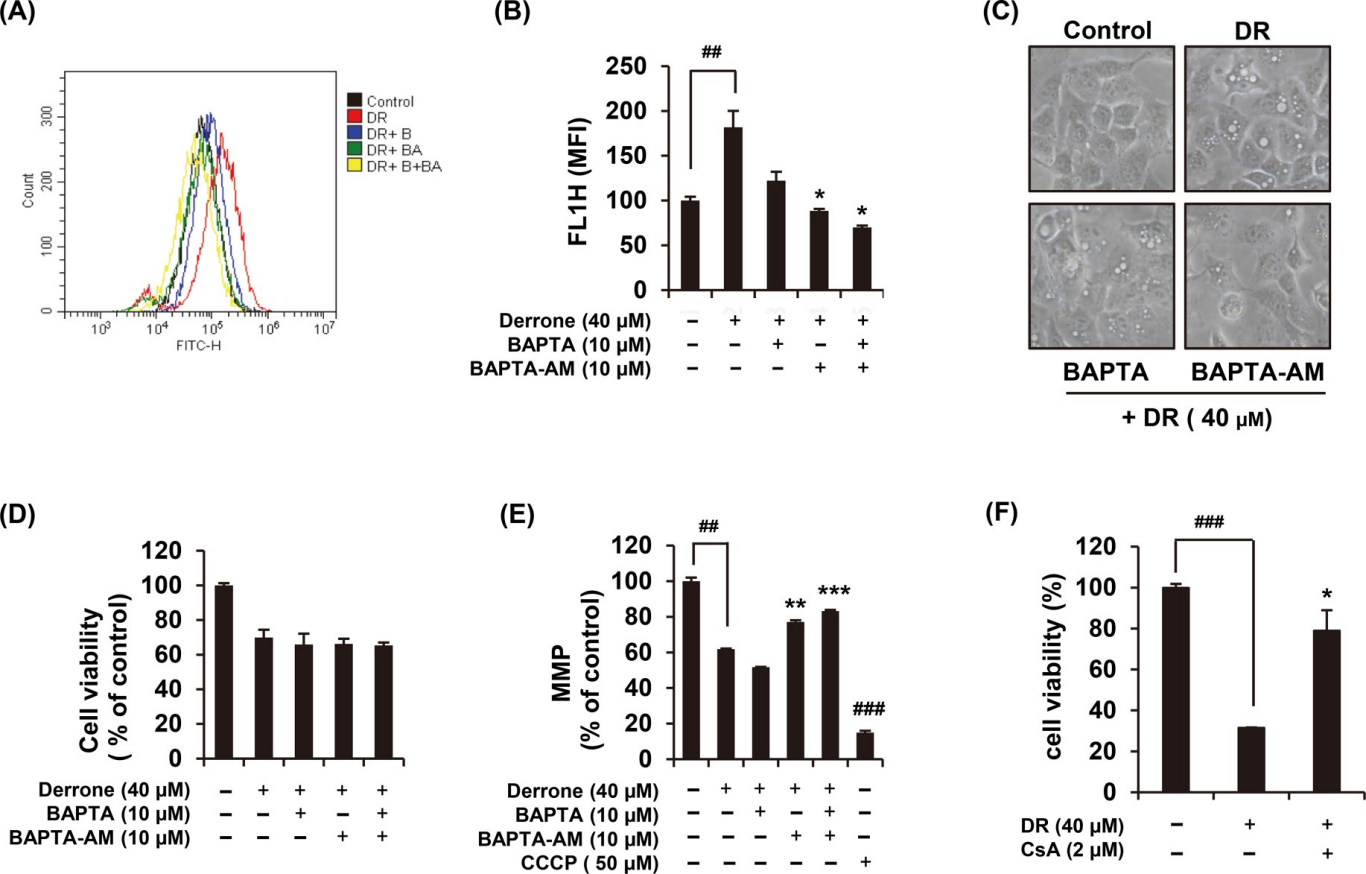
DR increases cytoplasmic Ca^2+^ concentration. (A) DR was added to 1 μg/ml Fluo-4 AM preloaded A549 cells and the changes of intracellular Ca^2+^ were examined by flow cytometry. Cells were stained with 1 μg/ml Fluo-4 AM for 30 min and treated 40 μM DR with or without co-treatment with 10 μM BAPTA and/or 10 μM BAPTA-AM for 1 min. (B) Cytoplasmic Ca^2+^ was quantified and represented as a bar graph. (C) The morphological change of cells was observed under the microscope. (D) Cell viability was measured using MTT assay. (E) The cells were stained with 40 nM DiOC_6_(3) for 30 min at 37°C, and then analyzed by flow cytometry. (F) Cells were treated 40 μM DR for 24 h with or without treatment with 2 μM cyclosporine A. The cell viability was measured by MTT assay. All data are expressed as the mean ± S.D. obtained from at least three independent experiments. Statistical differences were presented p<0.01 (**) and p<0.001 (***) compared with the DR alone; p<0.01 (##) and p<0.001 (###) compared with the DMSO control.

Since intracellular Ca^2+^ is widely recognized as an important modulator of autophagy, we next investigated whether DR perturbs intracellular Ca^2+^ homeostasis. When 40 μM DR treated in Fluo-4 AM-prestained A549 cells, a cell-permeable Ca^2+^ indicator dye, cytoplasmic Ca^2+^ was increased within seconds (Fig 5A and 5B). We explored the sources of increased Ca^2+^ after DR treatment using Ca^2+^ chelators including BAPTA (extracellular Ca^2+^ scavenger) and BAPTA-AM (cell-permeable acetoxymethyl ester Ca^2+^ chelator). Flow cytometry analysis presented that the increased cytoplasmic Ca^2+^ by DR was significantly blocked by only BAPTA-AM, indicating that the origin of cytoplasmic Ca^2+^ was derived from an intracellular reservoirs such as ER and mitochondria (Fig 5A). The autophagosome formation by DR treatment was greatly inhibited by BAPTA-AM (Fig 5C); however, pretreatment of BAPTA or BAPTA-AM did not prevent DR-induced cell death (Fig 5D). Many studies established that an abrupt increase in intracellular ROS or Ca^2+^ is one of the causes of MMP reduction. We found that DR-induced MMP loss was significantly restored by BAPTA-AM (Fig 5E). Excessive decrease of MMP in the cell leads to mitochondrial permeability transition (MPT) pore opening, we showed that DR-induced cell death was significantly restored by pretreatment with cyclosporine A, which inhibited cyclophillin D, one of the constituent proteins of MPT pore (Fig 5F). Cyclosporine A treated alone for 24 hours with the various concentrations showed the decrease the cell growth, and in the group treated with 2 μM, the viability was significantly reduced to about 70%. There is the evidences that cyclosporine A induces apoptosis of cancer cells [22, 23]. Although 2 μM cyclosporine A alone inhibited cell growth (S1 Fig), pretreatment reduced cell death by inhibiting the DR-induced opening of MPT pore. Collectively, treatment of A549 cells with DR rapidly increases intracellular free calcium concentration in a short period of time, which appears to contribute to cytoplasmic vacuole formation and MMP reduction. However, the calcium chelators could not restore DR-induced cell death.

### DR induces prolonged ERK phosphorylation

**Fig 6:**
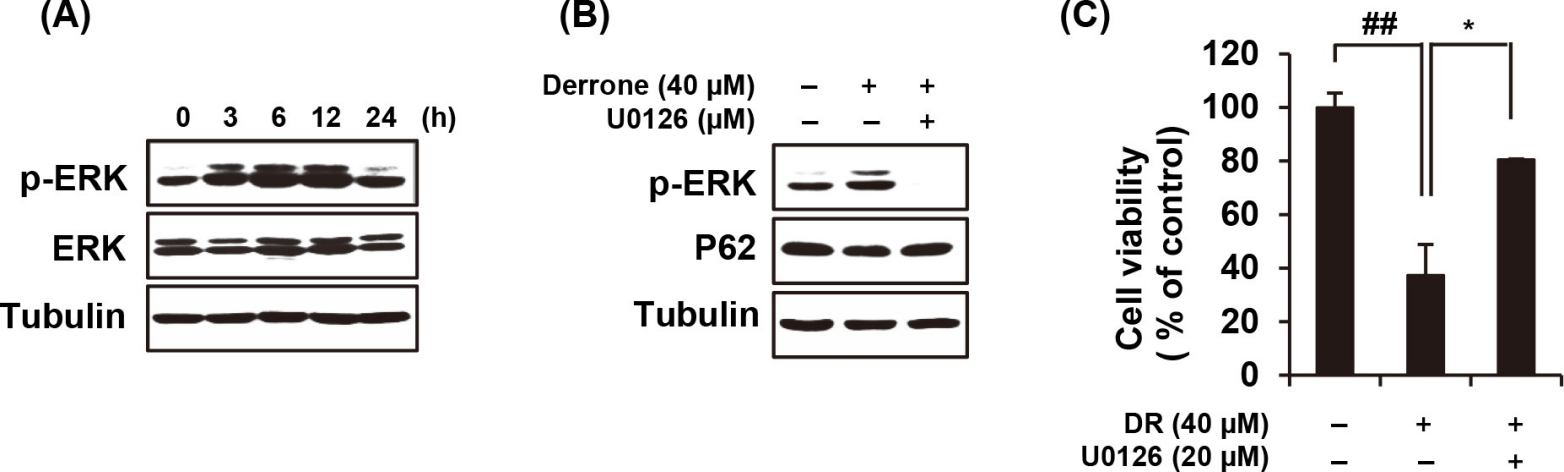
DR induces autophagy through ERK-mediated pathway. (A) After treatment of 40 μM DR for the indicated times, the A549 cells were lysated and detected p-ERK by western blotting. (B) Cells were pretreated with U0126 (ERK inhibitor) further treated with DR for 24 h. Western blotting of the indicated proteins was performed, with tubulin detected as a loading control. (C) Cell viability was analyzed MTT assay. All data are expressed as the mean ± S.D. obtained from at least three independent experiments. Statistical differences were considered significant if p<0.05 (*), p<0.01 (**), and p<0.001 (***) compared with the DR alone; p<0.01 (##) compared with the DMSO control.

Because the ERK plays a crucial roles in modulating autophagy [24], we have confirmed the increased phosphorylation of ERK by the treatment of DR. A continuous (about 12 h) increased p-ERK was observed when 40 μM DR treated (Fig 6A). The pretreatment of U0126, a highly selective inhibitor of both MEK1 and MEK2, markedly reversed the DR-mediated phosphorylation of ERK and degradation of p62 (Fig 6B). As illustrated in Fig 6C, the cell death induced by DR was effectively restored by U0126. Collectively, these results suggested that DR sustains ERK phosphorylation, which is closely related to DR-induced autophagic cell death.

## Discussion

In this study, we reported that DR, one of the major constituents of unripe fruits of *C*. *tricuspidata*, induces autophagic cell death as well as partial apoptosis in A549 cells.

Our results provide evidence that DR induces autophagy. First, an autophagosome surrounded by a double membrane was observed by electron microscope (Fig 3B). Second, the formations of LC3-II and decrease of p62 were confirmed by Western blot (Fig 3C). Third, pretreatment of CQ decreased the vacuole of cytoplasm increased by DR and restored decreased p62 expression (Fig 3D and 3F). Finally, we can demonstrate that DR induces autophagic cell death by restoring the DR-mediated cell death by CQ (Fig 3E).

The molecular mechanisms we have found in relation to DR-induced autophagic cell death in A549 cells are as follows. Treatment with DR increases intracellular ROS levels, which is highest at 1 hour after treatment (Fig 4A). Antioxidant agents significantly restored DR-mediated formation of vacuoles as well as cell death (Fig 4B and 4C), indicating that ROS production by DR is associated with autophagic cell death. Oxidative stresses caused by ROS can induce rapid depolarization of MMP [25]. Expectedly, we confirmed that DR induced depolarization of MMP and it was effectively recovered by NAC treatment (Fig 4D). In addition, DR increased the Ca^2+^ concentration in the cytoplasm in a second (Fig 5A and 5B), and BAPTA-AM, an intracellular Ca^2+^ chelator, significantly inhibited DR-induced formation of cytoplasmic vacuoles as well as reduction of MMP (Fig 5C and 5E). Although there have been several previous studies showing that cytoplasmic Ca^2+^ overload induces autophagy [26, 27], the detailed mechanism of DR-induced autophagy associated with Ca^2+^ need to be further explored. Finally, noteworthy is that prolonged activation of ERK signaling is required for DR-induced autophagy induction in A549 cells (Fig 6A). Inhibition of ERK activation by U0126 blocked autophagic cell death (Fig 6C), suggesting that ERK may acts as another upstream mediator of DR-induced autophagic cell death.

Constitutive activation of ERK by Raf [28], cadmium [29] or IGF-I receptor [30] has been reported to induce a cell death associated with cytoplasmic massive vacuolization. This morphology could be a sign of autophagic programmed cell death, but also of paraptosis, a form of caspase‐independent cell death associated with cytoplasmic vacuolization [31]. Therefore, we tried to determine whether the inhibition of ER stress with cycloheximide, which inhibited the protein synthesis by the newly synthesized intracellular proteins, and that it not affected DR-induced cell death (S2 Fig). More specifically, the fate of the cell depends on the degree of activation of ERK. Transient or moderate ERK activation increases cytoprotective autophagy through the increase of Beclin1 by inhibition of mTORC1 or mTORC2, however, sustained or strong ERK activation completely inhibits mTORC1 and mTORC2, leading to an explosive increase of Beclin1 leading to ultimate cytotoxic autophagy [32, 33]. In addition, in order to examine the effect of ROS scavenger or ERK inhibitor on autophagy, it was confirmed by Western blot that the DR-induced cleavage of LC3-I to LC3-II was suppressed after pretreatment of these compounds. The cleavage induced by DR was moderately reduced by the pretreatment of 20 μM U0126 and almost completely inhibited by pretreatment of NAC and GSH (S3 Fig). Many documents suggest that unusually prolonged ERK activation (between 6 and 72 h) associated with cell death requires the presence of ROS [34–36]. Our results also agree with it that the ROS induced by the derrone treatment may be crucial for inducing cell death.

A549 cells are p53 wild-type and therefore are highly resistant to DNA damaging agents such as cisplatin. A549 cells are actively used for studying resistance and recurrence of cancer, and studies using cancer stem cells derived from cisplatin-resistant A549 cells have been actively conducted. Of course, p53 mutations are observed in more than 50% of solid tumors. Therefore, we examined the cell viability of human non-small cell lung carcinoma cell line H1299 (p53-null) by treatment with DR with concentration-dependent manner for 24 h (S4 Fig). As a result, the IC_50_ value of DR in H1299 cells was 27.2 μM, and the IC_50_ value in A549 cells was 42.7 μM, which seems to be no significant difference in IC_50_ values. The results of treatment with DR in the H1299 (p53-null) cells were unexpectedly different from the mode of action in A549 cells. First, a form of DR-mediated cell death in H1299 cells is different from that of A549 cells. In H1299 cells, there was no recovery of DR-induced cell death by pretreatment of the autophagy inhibitors such as chloroquine or wortmannin (S5A Fig). In addition, DR-mediated cell death in H1299 cells was not restored by treatment with the ERK inhibitors U0126 or PD98059 (S5B Fig). Western analysis confirmed that DR-mediated induction of ERK phosphorylation and cleavage from LC3-I to LC3-II in H1299 cells. Pretreatment of U0126 significantly inhibited DR-mediated ERK phosphorylation and cleavage from LC3-I to LC3-II, but ultimately did not inhibit DR-induced autophagic cell death (S5C Fig). Previous evidences have shown that ERK affects p53 stability and activity, and may affect autophagy and cell death modes [31]. Taken together, DR induces autophagy in p53-null cells but does not contribute to cell death, and whether or not p53-dependent apoptosis contributes to DR-mediated cell death should be clarified in future studies.

As far as cancer is concerned, autophagy can act as a tumor suppressor and tumor promoter [37–39]. Decreased autophagy promotes tumorigenesis because monoallelical loss of essential autophagy gene ATG6/BECN1 in 40–75% of in human prostate, breast, and ovarian cancers is observed [39–41]. Autophagy inducers may activate apoptosis-independent cell death in apoptosis-resistant cells lacking the expression of the pro-apoptotic proteins such as Bax and Bak [42, 43]. This suggests that continuous research on the autophagy inducer is necessary from the viewpoint of replacing the apoptosis inducer used as a conventional anticancer chemotherapeutic agent. Indeed, several chemotherapeutic drugs have been reported to engage autophagy [37, 44, 45].

Our previously research has shown that unripe fruits contains different constituents compared to ripe fruits, which resulted in the excellent biological activities. In this study, we have provided evidence indicating that DR induces autophagic cell death through intracellular ROS and sustained ERK phosphorylation in A549 cells. Therefore, DR from unripe fruits of *C*. *tricuspidata* may act as a natural bioactive substance that was chemotherapeutic drugs through the activation of autophagy.

## Supporting information

S1 Table 1H NMR data of derrone (methanol-*d*_4_, 500 MHz). (PDF)

S1 Fig Effect of cyclosporine A alone on cell viability of A549 cells. Cells were treated with the cyclosporine A of indicated concentrations for 24 h, and then measured by WST assay. Differences were considered significant at p<0.05 (*) compared with the DMSO control.
(PDF)

S2 Fig Effects of pretreatment of cycloheximide on DR-induced cell death in A549 cells. Cells were co-treated with CHX and 40 μM DR for 24 h and then measured by WST assay. Statistical differences were presented p<0.001 (###) compared with the DMSO control.
(PDF)

S3 Fig Effect of ROS scavenger or ERK inhibitor on DR-induced autophagy. Cells were pretreated with U0126, NAC and GSH, and further treated with DR for 24 h. Cells were lysated and detected LC3 by western blotting. The tubulin detected as a loading control.
(PDF)

S4 Fig Effect of DR on cell growth of H1299 cells. H1299 cells were treated with the DR with indicated concentrations for 24 h, and then measured by MTT assay. Differences were considered significant at p<0.05 (*) and p< 0.01 (**) compared with the DMSO control.
(PDF)

S5 Fig The autophagy and ERK associated with DR-induced cell death in H1299 cells. (A and B) H1299 cells were pre-treated with chloroquine, wortmannin, U0126 or PD98059, and exposed 40 μM DR further 24 h. Cell viability was measured by MTT assay. (C) H1299 cells were treated with DR with or without U0126 for 24 h. Western blotting was performed to detect p-ERK, ERK, LC3 and Tubulin.
(PDF)
