# Supplementary material for: Derrone induces autophagic cell death through induction of ROS and ERK in A549 cells
Source: PLoS One. 2019 Jun 19;14(6):e0218659. doi: 10.1371/journal.pone.0218659 (PMC6583947; doi:10.1371/journal.pone.0218659)
Supplement: S1 Table — (PDF) [file pone.0218659.s001.pdf]

S1 Table. 1H NMR data of derrone (methanol-d_4, 500 MHz).

Carbon NO.	derrone
	^{1}H (M, 500MHz)
1	-
2	8.19 (1H, s)
3	-
4	-
4a	-
5	-
6	6.23 (1H, s)
7	-
8	-
8a	-
1'	-
2'	7.42 (1H, d, *J* = 8.5)
3'	6.87 (1H, d, *J* = 8.5)
4'	-
5'	6.87 (1H, d, *J* = 8.5)
6'	7.42 (1H, d, *J* = 8.5)
1''	6.76 (1H, d, *J* = 10.5)
2''	5.73 (1H, d, *J* = 10.0)
3''	-
4''	1.49 (3H, s)
5''	1.49 (3H, s)
