# Supplementary figures and images for: Derrone induces autophagic cell death through induction of ROS and ERK in A549 cells
Source: PLoS One. 2019 Jun 19;14(6):e0218659. doi: 10.1371/journal.pone.0218659 (PMC6583947; doi:10.1371/journal.pone.0218659)

**S1 Fig**

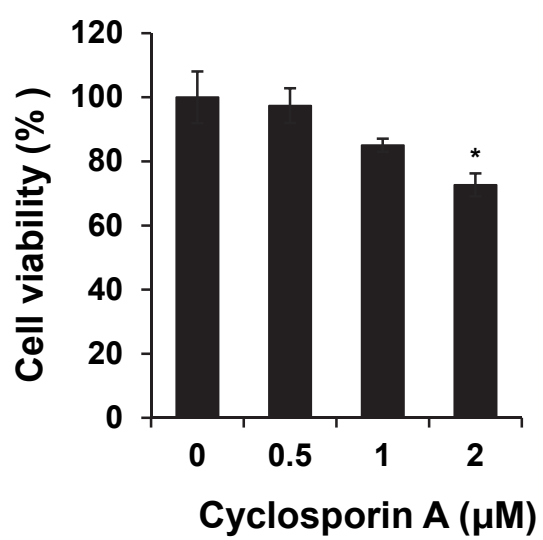

Supplement: S1 Fig — Cells were treated with the cyclosporine A of indicated concentrations for 24 h, and then measured by WST assay. Differences were considered significant at p<0.05 (*) compared with the DMSO control. (PDF) [file pone.0218659.s002.pdf]

**S2 Fig**

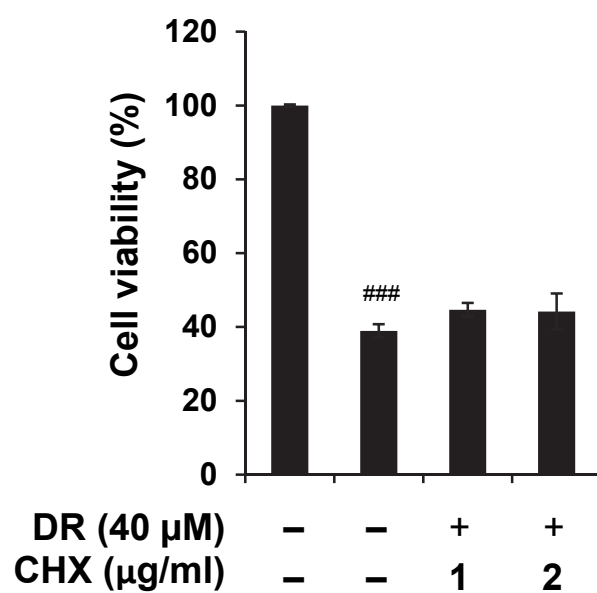

Supplement: S2 Fig — Cells were co-treated with CHX and 40 μM DR for 24 h and then measured by WST assay. Statistical differences were presented p<0.001 (###) compared with the DMSO control. (PDF) [file pone.0218659.s003.pdf]

## S3 Fig

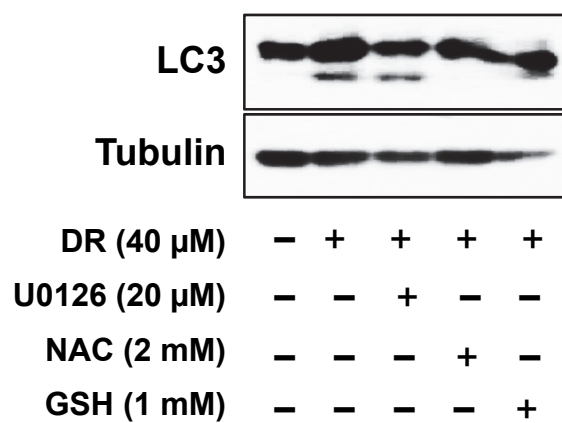

Supplement: S3 Fig — Cells were pretreated with U0126, NAC and GSH, and further treated with DR for 24 h. Cells were lysated and detected LC3 by western blotting. The tubulin detected as a loading control. (PDF) [file pone.0218659.s004.pdf]

**S4 Fig**

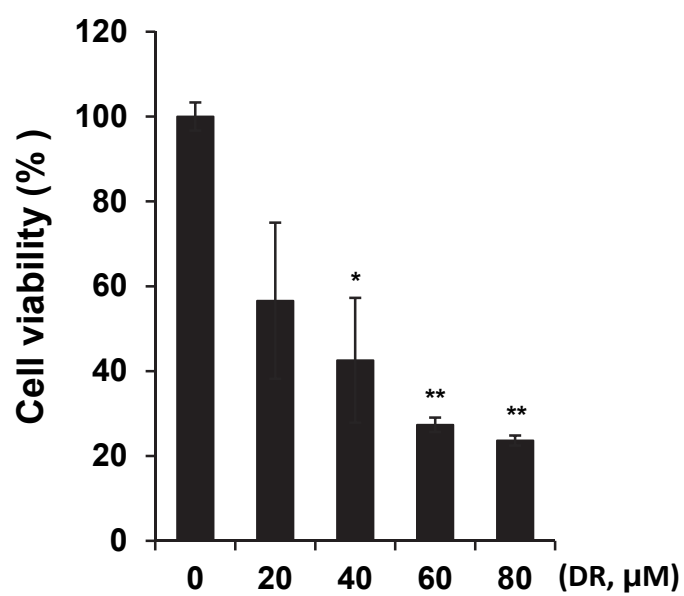

Supplement: S4 Fig — H1299 cells were treated with the DR with indicated concentrations for 24 h, and then measured by MTT assay. Differences were considered significant at p<0.05 (*) and p< 0.01 (**) compared with the DMSO control. (PDF) [file pone.0218659.s005.pdf]

# S5 Fig

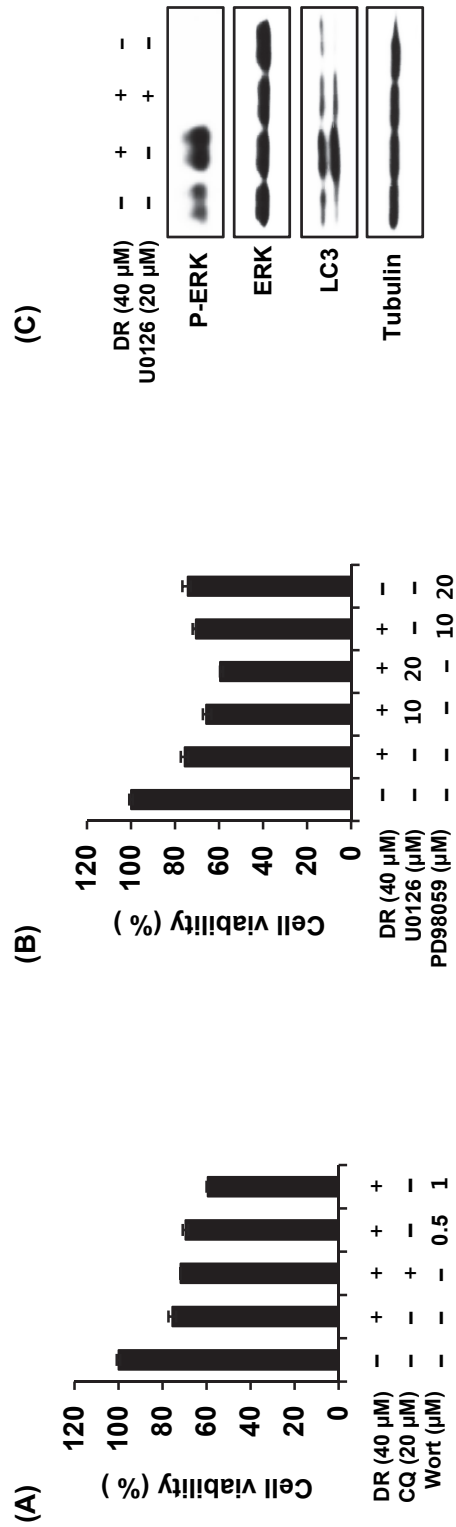

Supplement: S5 Fig — (A and B) H1299 cells were pre-treated with chloroquine, wortmannin, U0126 or PD98059, and exposed 40 μM DR further 24 h. Cell viability was measured by MTT assay. (C) H1299 cells were treated with DR with or without U0126 for 24 h. Western blotting was performed to detect p-ERK, ERK, LC3 and Tubulin. (PDF) [file pone.0218659.s006.pdf]
